# TCRLens: structure-aware equivariant graph learning for TCR-pMHC-I recognition and immunogenic epitope discovery

**DOI:** 10.1093/bioadv/vbag066

**Published:** 2026-02-24

**Authors:** Paopit Siriarchawatana, Supawadee Ingsriswang, Challika Kaewborisuth, Anan Jongkaewwattana

**Affiliations:** Microbial Systems and Computational Biology Research Team, Thailand Bioresource Research Center (TBRC), Pathum Thani, 12120, Thailand; National Center for Genetic Engineering and Biotechnology (BIOTEC), Pathum Thani, 12120, Thailand; Microbial Systems and Computational Biology Research Team, Thailand Bioresource Research Center (TBRC), Pathum Thani, 12120, Thailand; National Center for Genetic Engineering and Biotechnology (BIOTEC), Pathum Thani, 12120, Thailand; National Center for Genetic Engineering and Biotechnology (BIOTEC), Pathum Thani, 12120, Thailand; Virology and Vaccine Technology Research Team, Veterinary Health Innovation and Management Research Group, Pathum Thani, 12120, Thailand; National Center for Genetic Engineering and Biotechnology (BIOTEC), Pathum Thani, 12120, Thailand; Virology and Vaccine Technology Research Team, Veterinary Health Innovation and Management Research Group, Pathum Thani, 12120, Thailand

## Abstract

**Motivation:**

Accurate prediction of T-cell receptor (TCR) recognition of peptide-MHC class I (pMHC-I) complexes is a key challenge due to structural diversity and data sparsity. We introduce TCRLens, a structure-aware deep learning framework that models residue-level interactions across five critical interface zones using multi-scale graph representations and an equivariant graph neural network (EGNN). To mitigate data sparsity and severe class imbalance arising from limited negative samples, TCRLens incorporates a variational autoencoder-generative adversarial network (VAE-GAN) to generate structurally plausible weak-affinity interaction samples. We evaluated TCRlens across three prediction tasks including peptide-MHC binding, peptide-TCR recognition, and full-complex TCR-pMHC-I interaction and observed consistently strong performance.

**Results:**

Using curated dataset of human TCR-pMHC-I structural complexes from ATLAS and TCR3d, TCRLens outperforms state-of-the-art sequence-based, motif-based, and structure-aware methods, including NetMHCpan 4.2, CapsNet-MHC, RPEMHC, NetTCR-2.0, TITAN, PanPep, pMTnet, ERGO II, and STAG. Furthermore, TCRLens demonstrates robust cross-species generalization, achieving high predictive performance in swine and chicken MHC-I systems. These findings highlight the value of geometry-aware representation learning and generative data augmentation for capturing immunological specificity. TCRLens provides a unified and extensible platform for TCR-pMHC-I interaction modeling, with potential applications in epitope discovery and structure-guided vaccine design across both human and veterinary immunology.

**Availability and implementation:**

The code used in this study is publicly available at https://github.com/paopitsiri/TCRLens.

## 1. Introduction

The TCR-pMHC-I interaction, defined as the molecular recognition between T-cell receptors (TCRs) and peptide-major histocompatibility class I complex (pMHC-I) molecules, is a cornerstone of adaptive immunity. It enables T cells to distinguish between self and non-self peptides presented on the surface of antigen-presenting cells, thereby underpinning the specificity of T-cell-mediated immune responses (Shevyrev *et al.* 2022). Beyond its central role in immunosurveillance, this interaction forms the basis of numerous immunotherapeutic strategies, including peptide- and neoantigen-based vaccine design (Rudolph *et al.* 2006). Despite its importance, accurately predicting TCR-pMHC-I binding remains a significant challenge due to the immense diversity of TCR repertoires, extensive MHC polymorphism and the structural complexity of the binding interface.

TCR-pMHC-I recognition involves three coupled interaction modes: peptide-MHC, peptide-TCR, and MHC-TCR interactions (Yin *et al.* 2012) (Supplementary Fig. S1). While antigenic specificity is largely determined by the highly variable complementarity-determining region 3 (CDR3) loops of the TCR, the more conserved CDR1 and CDR2 loops mainly engage the MHC α-helices, contributing to MHC restriction and docking geometry (Piepenbrink *et al.* 2013). Even subtle polymorphisms among MHC class I alleles can reshape the peptide-binding groove, thereby modulating both peptide conformation and altering the TCR-contacting surface. Accurately modeling these structurally distinct components is therefore critical for understanding TCR specificity and cross-reactivity.

A wide range of computational approaches have been proposed to model different aspects of this recognition process. Sequence-based peptide-MHC binding predictors such as NetMHCpan 4.2 (Nilsson *et al.* 2025), CapsNet-MHC (Kalemati *et al.* 2023) and RPEMHC (Wang *et al.* 2024) focus on antigen presentation, while TCR-centric models including NetTCR-2.0 (Montemurro *et al.* 2022), PanPep (Gao *et al.* 2023), and TITAN (Weber *et al.* 2021) learn peptide-TCR binding preferences primarily driven by CDR3 sequence features. More integrative deep learning frameworks, such as ERGO-II (Springer *et al.* 2021), TCRpeg (Jiang and Li 2023), and DeepTCR (Sidhom *et al.* 2021) attempt to model the full TCR-peptide-MHC triad. However, most existing methods rely predominantly on sequence-level representations and lack explicit structural and physicochemical context. In particular, interactions involving MHC residues and TCR CDR1/CDR2 loops are often underrepresented or implicitly modeled, limiting interpretability and predictive robustness (Attaf *et al.* 2016).

Recent structure-aware approaches have begun to address these limitations. GraphMHC (Jeong *et al.* 2024) demonstrated the potential of graph-based structural representations for peptide-MHC modeling, while STAG (Slone *et al.* 2025) introduced graph neural networks for TCR-pMHC interaction analysis. Nevertheless, many existing models primarily focus on peptide-mediated contacts in modeling TCR recognition. As a result, MHC-TCR interactions beyond CDR3—critical determinants of binding stability and signaling, are insufficiently captured, preventing a complete representation of the interaction interface.

From an evolutionary perspective, the three-dimensional (3D) structural fold of MHC class I molecules is highly conserved across vertebrates, including humans, pigs, and chickens (Koch *et al.* 2007, Kaufman 2018, Youk *et al.* 2023). This conserved architecture, comprising an α1–α2 peptide-binding groove supported by an α3 domain and β2-microglobulin, enabled broadly similar antigen presentation mechanisms across species. In contrast, TCR repertoire shows more structural divergence, particularly in constant domain architecture and CDR loop configurations. Mammalian TCRs, such as those in Sus scrofa, closely resemble human TCRs in V(D)J gene usage and 3D conformation (Butler *et al.* 2006, Wang *et al.* 2017, Yang *et al.* 2019), suggesting that models trained on human data may generalize across mammals. Avian TCRs, exemplified by *Gallus gallus*, exhibit distinct structural features including a shortened Cβ FG loop and extended Cα DE loop, which may require species-specific modeling strategies (Zhang *et al.* 2020). These observations highlight both the feasibility and challenges of cross-species TCR-pMHC-I modeling.

**Figure 1:**
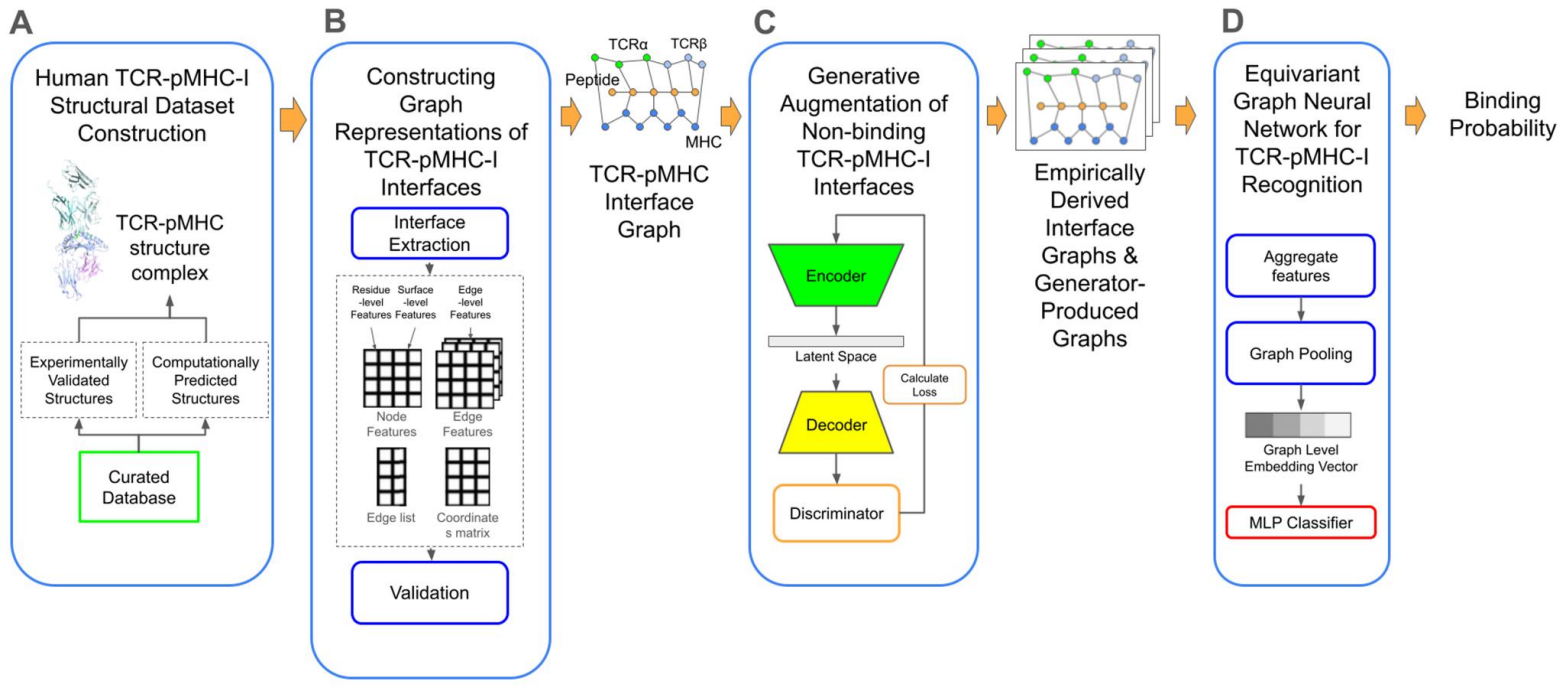
Overview of the TCRLens workflow. (A) Construction of the human TCR-pMHC-I structural dataset. (B) Graph-based representation of TCR-pMHC-I interfaces. (C) Generative augmentation of weak-binding TCR-pMHC-I interfaces. (D) Equivariant graph neural network architecture for TCR-pMHC-I recognition.

To address these limitations, we introduce TCRLens, a structure-aware, graph-based deep learning framework for TCR-pMHC-I binding prediction (Fig. 1). TCRLens integrates three core components: (i) multi-level structural representation, (ii) generative data augmentation, and (iii) equivariant graph learning. The structural representation encodes interaction features at residue-, edge-, and surface-level resolutions, enabling simultaneous modeling of fine-grained atomic contacts and global interface geometry. Using high-resolution TCR-pMHC-I complexes curated from experimental and computational structural databases, we extract interface contacts spanning five biologically relevant zones: peptide-MHC, peptide-TCRα, peptide-TCRβ, MHC-TCRα, and MHC-TCRβ.

Each complex is represented as a graph, where nodes correspond to contacting residues, edges capture spatial or chemical relationships, and surface-level features describe residue exposure and burial within the interface. Graphs are enriched with interpretable physicochemical and structural attributes, including amino acid identity, inter-residue distances, electrostatic and van der Waals potentials, solvent accessibility and residue burial. Unlike STAG, which represents node properties using abstract amino acid embeddings such as Atchley (Atchley *et al.* 2005) or Kidera (Kidera *et al.* 1985) factors, TCRLens explicitly models all cross-chain contacts using physically grounded descriptors, enabling a more complete and interpretable characterization of the interaction landscape.

At the core of TCRLens is an Equivariant Graph Neural Network (EGNN) (Satorras *et al.* 2021), which jointly updates node features and 3D coordinates while preserving rotational and translational equivariance. This geometry-aware message-passing scheme allows the model to learn spatially grounded interaction patterns that generalize across unseen interfaces and diverse MHC alleles. To mitigate the scarcity of experimentally validated weak-affinity binding structures and associated class imbalance, TCRLens incorporates a generative augmentation module based on a variational autoencoder-generative adversarial network (VAE-GAN) (Sainburg *et al.* 2018). This module learns latent representations of interface graphs and generates structurally coherent decoy (low-affinity binding) complexes by perturbing interaction features, thereby augmenting negative samples and improving robustness in low-data and cross-domain settings.

For affinity binding prediction, both experimentally derived (extracted from TCR-pMHC structures) and VAE-GAN-generated graphs are processed through the EGNN to produce refined residue-level embeddings. The resulting graph-level representations are passed through a multilayer perceptron (MLP) to estimate TCR-pMHC-I binding probability. A predefined decision threshold is applied to the predicted probabilities to yield a binary classification, assigning each complex as either strong-binding or weak-binding interaction. This end-to-end architecture allows TCRLens to capture the structural and biophysical complexity of immune recognition while compensating for limitations in available training data.

We validated TCRLens across multiple benchmarks, including residue-level contact prediction and binding classification tasks. The framework consistently outperformed sequence-based models (NetMHCpan 4.2, CapsNet-MHC, RPEMHC, NetTCR-2.0, PanPep, TITAN) as well as integrative approaches such as ERGO-II, pMTnet, and STAG across peptide-MHC, peptide-TCR, and pan-specific TCR-pMHC-I settings. In addition, TCRLens maintained strong predictive performance in cross-species evaluations involving *Sus scrofa* and *Gallus gallus*, highlighting its capacity to generalize beyond human immune systems. Together, these results establish geometry-aware modeling of the complete TCR-pMHC-I interface as a powerful and biologically grounded paradigm for immune recognition prediction.

## 2. Methods

### 2.1. Human TCR-pMHC-I structural dataset construction

We assembled a curated dataset of human TCR-pMHC-I structural complexes for model training, validation, and testing as outlined in Fig. 1A. Experimentally resolved three-dimensional (3D) structures were obtained from ATLAS (Borrman *et al.* 2017) and TCR3d (Gowthaman and Pierce 2019). Duplicate TCR-peptide-MHC complexes arising from overlaps between the ATLAS and TCR3d databases were excluded. In addition, complexes lacking experimentally measured binding affinity information (Kd) were excluded. The complete list of TCR-pMHC-I complexes and their corresponding affinity annotations extracted from the ATLAS is provided in Supplementary Table S5. To expand epitope-TCR coverage beyond available crystal structures, complexes lacking structural data were modeled using the tFold-TCR pipeline (Wu *et al.* 2025) and incorporated into the dataset.

The final curated dataset comprised 620 TCR-pMHC-I complexes, including 463 experimentally determined structures and 158 computationally predicted structures. Among the experimentally resolved complexes, 392 were annotated as strong-binding interactions and 71 as weak-binding interactions, while the predicted set contained 150 strong-binding and 7 weak-binding complexes. All complexes were standardized by enforcing consistent peptide orientation and verifying chain assignments for TCRα, TCRβ, the MHC heavy chain, and the bound peptide.

Binding labels were assigned using reported dissociation constants (Kd), which were experimentally measured in solution using purified proteins, typically via surface plasmon resonance (SPR) or isothermal titration calorimetry (ITC) as curated in the ATLAS and TCR3d databases: complexes with Kd < 200 µM were classified as strong-binding interactions, whereas those with Kd > 200 µM were designated as weak-binding interactions. This threshold of Kd is consistent with prior studies indicating that functional TCR-pMHC interactions typically exhibit affinities in the 1–200 µM range, and while interactions exceeding ∼200 µM are weak, transient and rarely detectable by conventional pMHC tetramer assays (Stone *et al.* 2009, Dolton *et al.* 2014, Hoffmann and Slansky 2020).

The resolution of experimentally determined structures ranged from 1.8 Å to 3.8 Å (mean 2.64 Å, median 2.60 Å), corresponding to high- to medium-resolution X-ray crystallographic data. All retained complexes were further processed to identify interface residues and construct residue-level interaction graphs, which served as inputs for downstream geometry-aware learning.

### 2.2. Constructing graph representations of TCR-pMHC-I interfaces

To model the structural interface of TCR- pMHC-I complexes, we constructed residue-level interaction graphs using the DeepRank2 framework (Crocioni *et al.* 2024) (Fig. 1B). For each complex, interface residues were extracted and represented as an undirected graph. Nodes correspond to individual interface residues from the TCR, the peptide, and the MHC components and were annotated with multiple feature classes, including (i) amino-acid identity encoded as a 20-dimensional one-hot vector, (ii) physicochemical descriptors such as charge, hydrophobicity, polarity, and side-chain volume, (iii) interface and interaction-related features, and (iv) 3D residue coordinates with associated backbone geometric features. All continuous features, encompassing physicochemical, interface, and geometric feature (ii–iv), were normalized prior to model training. Edges represent residue-residue interactions and were defined between residues within an 8 Å cutoff, measured between heavy side-chain atoms. Edge features captured both geometric and physicochemical interaction properties, including inter-residue geometric distances, orientation-dependent angular descriptors, and binary indicators of specific interaction types such as hydrogen bonds, salt bridges, or hydrophobic contacts.

Interactions were extracted across five biologically relevant interfaces: MHC-peptide, peptide-TCRα, peptide-TCRβ, MHC-TCRα, and MHC-TCRβ—which were initially represented as separate graphs and subsequently merged into a unified graph capturing the full TCR-pMHC-I interface. During graph merging, nodes with identical chain identifiers and residue indices were consolidated, and redundant edges were removed, yielding a single non-redundant representation. The unified graph was used for all model training and downstream analyses, whereas interface-specific graphs were retained only for intermediate processing. All graph data were stored in HDF5 format (Folk *et al.* 2011), with each entry containing graph metadata along with matrices encoding node and edge-level features. A summary of selected features is provided in Supplementary Table S1.

To evaluate whether TCR-pMHC-I structures predicted by tFold-TCR preserve interaction patterns observed in experimentally resolved complexes, we performed a pairwise comparative analysis between experimental and predicted models. For each experimentally determined complex, a corresponding predicted structure was generated using tFold-TCR, from the same TCR and pMHC-I amino acid sequences. Both experimental and predicted structures were processed identically using DeepRank2 to ensure consistent interface graph construction.

Complementarity-determining regions (CDRs) of TCR α and β chains were annotated using ANARCI (Dunbar and Deane 2016) with IMGT-based numbering (Lefranc *et al.* 2003), enabling standardized identification of CDR1, CDR2, and CDR3 boundaries. This annotation facilitated residue-level correspondence between experimental and predicted structures. Subgraphs corresponding to CDR-peptide and CDR-MHC interactions were then extracted by selecting edges linking CDR residues to contacting pMHC residues. Protein-protein interfaces and residue-level contacts were further validated using PISA (Krissinel and Henrick 2007), ensuring that extracted interactions corresponded to physically plausible TCR-pMHC-I interface regions.

For each of six CDR loops (α1–3 and β1–3), we quantified three interaction metrics: (i) the mean Euclidean distance between CDR residues and their peptide or MHC contacts, (ii) the number of contacting peptide or MHC residues, and (iii) the number of contacting CDR residues. These metrics were computed separately for both the experimental and predicted structures, and their distributions were compared using a two-sided Mann-Whitney *U* test and quantified using Cliff’s delta, to evaluate whether predicted interfaces exhibited statistically significant deviations from experimentally resolved interactions.

### 2.3. Generative augmentation of weak-binding TCR-pMHC-I interfaces

To augment the training set and enhance discrimination between high-affinity and weak-affinity TCR-pMHC-I interactions, we developed a generative framework based on a Graph Variational Autoencoder combined with a Generative Adversarial Network (Graph VAE-GAN) as illustrated in Fig. 1C. This framework generates structure-guided decoy interface graphs of TCR-pMHC-I complexes, specifically enriching the dataset with weak-affinity binding complexes that correspond to energetically unfavorable or geometrically inconsistent interface configurations, representing weak-affinity interaction states.

In contrast to conventional VAE-GAN architectures optimized for image or fixed-length sequential data, our framework is specifically designed for variable-sized, graph-structured representations of biological interfaces. It jointly models residue-level (node) and interaction-level (edge) features, enabling detailed reconstruction of both spatial geometry and physicochemical interaction patterns. To accommodate heterogeneity in interface sizes, we implement size-specific decoders, circumventing the fixed-dimensional constraints of standard generative models. The discriminator operates on a vectorized representation of the graph, produced by flattening the concatenated adjacency and node feature matrices, allowing it to capture fine-grained structural deviations. These architectural adaptations enable the generation of structurally plausible yet weak-binding TCR-pMHC-I decoys, synthetic negatives that are difficult to produce using conventional generative approaches.

The Graph VAE-GAN architecture consists of three core components:

- Encoder: A two-layer Graph Convolutional Network (GCN) that encodes the input TCR-pMHC-I interface graph into a latent representation. The encoder processes both node and edge features, embedding the edge attributes and integrating them with node-level information. A global pooling operation aggregates graph-level features, and the network outputs the mean and log-variance vectors of a multivariate Gaussian, enabling stochastic sampling of latent codes that capture the diversity of real binding interfaces.
- Decoder: A graph generator that reconstructs biologically plausible interface graphs from latent vectors. It predicts the adjacency matrix, node features, and edge features to closely match real TCR-pMHC-I graphs. To accommodate variability in graph sizes (i.e. number of residues per complex), the architecture employs separate decoders for each graph size category, enhancing scalability and structural fidelity.
- Discriminator: Wasserstein GAN (Arjovsky *et al.* 2017): A discriminator network trained using Wasserstein distance with gradient penalty to distinguish empirically derived interface graphs from generator-produced graphs. It operates on a flattened representation that concatenates the adjacency matrix, node, and edge features. Regularization techniques including weight clipping and noise injections are used to improve training stability and adversarial robustness. The discriminator guides the generator to produce structurally plausible yet functionally weak-binding decoy graphs.

The Graph VAE-GAN was trained exclusively on weak-affinity TCR-pMHC-I interface graphs derived from experimentally characterized weak-binding complexes curated from the ATLAS and TCR3d datasets. The model was optimized to learn the empirical distribution of weakly interacting interfaces, thereby expanding under-represented regions of the structural interaction space while preserving physicochemical consistency and geometric plausibility, without bias toward binder-like or energetically favorable interface topologies. Hyperparameters were selected through manual tuning based on performance on a held-out validation subset derived from the training data. Candidate configurations were evaluated based on stability of adversarial training, convergence of reconstruction loss, and preservation of physically plausible interface geometries. The final model employed a latent dimensionality of 16 and a hidden dimensionality of 64, with learning rates of 1 × 10^−3^ for the encoder-decoder and 1 × 10^−4^ for the discriminator. Adversarial training was performed using Wasserstein GAN objective with weight clipping, with an optional feature-matching term to stabilize training. Early stopping was applied based on convergence of the reconstruction loss.

Next, to assess the quality of the generated weak-affinity binding samples, we employed a multi-level evaluation strategy comprising feature-level, topology-level, and latent-space-based criteria.

- Local distributional similarity between real and generated graphs was measured using the Kullback-Leibler (KL) divergence (Kullback and Leibler 1951), computed independently for individual node- and edge-level features, including residue polarity, solvent accessibility, residue mass, inter-residue distance, and electrostatic potential.
- Global structural coherence was evaluated using graph-level topological metrics, including connectivity, degree distribution, and clustering coefficient. Additionally, the Maximum Mean Discrepancy (MMD) (Sun and Fan 2024) between empirically derived and generated graphs was computed using the Weisfeiler-Lehman subtree kernel (Shervashidze *et al.* 2011), which provides a topology-aware measure of graph similarity.
- Latent space separation was assessed using graph-level embeddings learned by the Graph VAE-GAN. Generated samples were required to lie outside the high-density region of the latent space corresponding to experimentally validated strong-binding complexes. Decoy graphs exhibiting substantial overlap with the strong-binding interactions embedding distribution were excluded, thereby retaining weak-binding samples that occupy distinct regions of the learned interface manifold and reducing the likelihood of including binder-like or high-affinity complexes.

During training, stochastic components inherent to the variational and adversarial framework, such as latent sampling and randomized mini-batch optimization were enabled as required for effective learning. In contrast, during inference and evaluation, deterministic behavior was explicitly enforced by fixing random seeds, disabling stochastic layers, and using fixed latent representations. Under these conditions, repeated inference runs using identical model checkpoints and inputs produced identical generated graphs and evaluation metrics, ensuring full reproducibility despite the use of a variational architecture.

In downstream tasks, incorporating VAE-GAN-generated weak-affinity binding samples during EGNN training. In this setting, the 400 structure-guided decoys complexes that are weak-binding were generated to approximately balance the number of strong-binding and weak-binding samples, increasing diversity and structural challenge within the training set. Incorporating these decoys led to improved classification performance, particularly in settings involving high structural similarity between strong-binding and weak-binding interfaces. This resulted in measurable gains in AUC, precision, recall performance, and reduction in false positive predictions, supporting the effectiveness of generative augmentation in training robust structure-aware models for TCR-pMHC-I recognition.

### 2.4. Equivariant graph neural network for TCR-pMHC-I recognition

To capture both spatial and physicochemical properties of the TCR-pMHC-I interface, we implemented an Equivariant Graph Neural Network (EGNN) using the EGNN_Sparse module from the EGNN-Pytorch (Satorras *et al.* 2021) library. as illustrated in Fig. 1D and Supplementary Fig. S2. The EGNN was integrated into the DeepRank2 pipeline by replacing the default GNN layers with equivariant message-passing layers that operate directly on residue-level 3D coordinates, enabling rotation and translational equivariance in modeling interface interactions.

Each TCR-pMHC-I complex is represented as a residue-level graph, where nodes correspond to interface residues annotated with physicochemical and surface-derived features, and edges represent spatially proximal residue pairs with associated interaction features. For each node, the initial input to the EGNN consists of its residue-level feature vector together with the associated three-dimensional coordinates, while edge features encode geometric and energetic descriptors of residue-residue interactions.

The network architecture comprises two stacked EGNN layers, followed by a graph-level multilayer perceptron (MLP) classifier. Within each EGNN layer, equivariant message passing is carried out through a sequence of geometry-aware operations. First, relative displacement computation is performed for each connected residue pair by calculating relative displacement vectors and squared Euclidean distances from the three-dimensional coordinates. Second, message generation is achieved by combining node features, edge features, and relative geometric information into edge-specific messages using a shared multilayer perceptron. Third, equivariant coordinate update is applied by aggregating weighted displacement vectors from neighboring residues to update residue coordinates in a manner consistent with rigid-body transformations. Finally, message aggregation and node update are performed by aggregating incoming messages at each node and applying nonlinear transformations to produce updated node embeddings that encode the local structural context. This joint sequence of operations enforces equivariance at both the node and coordinate levels, enabling the model to learn geometry-aware interaction patterns within the TCR-pMHC-I interface.

Importantly, binding prediction is performed at the graph level rather than the node level. After equivariant message passing, the updated node embeddings are aggregated using mean pooling (scatter_mean in PyTorch Scatter; Fey and Lenssen 2019) to obtain a fixed-dimensional graph-level representation that summarizes the global structural and physicochemical characteristics of the TCR-pMHC-I interface. This pooling operation removes dependence on node ordering and yields an invariant representation with respect to rotations and translations of the input structure.

The resulting graph embedding is passed through a two-layer MLP with Rectified Linear Unit (ReLU) activations to produce a score output between 0 and 1, interpreted as the probability of binding. A threshold of .5 is applied to obtain a binary prediction (strong binding versus weak-binding). Thus, while equivariant learning is performed at the residue level, the final classification outcome is invariant and defined at the level of the entire TCR-pMHC-I complex.

Model hyperparameters were tuned using stratified validation splits within the training folds. A predefined set of candidate hyperparameters, including learning rate, hidden dimension size, number of EGNN layers, and dropout rate, was systematically evaluated within each training fold. The optimal configuration was selected based on validation AUC and loss stability, and the final model was then evaluated using 10-fold cross-validation. Models were trained for up to 100 epochs with a batch size of 8 using stochastic gradient descent (learning rate 1 × 10^−3^, weight decay 1 × 10^−3^). Early stopping was applied based on validation loss to reduce overfitting. To address class imbalance, training employed a weighted cross-entropy loss defined over three classes: experimentally validated binding interactions, experimentally validated weak-binding interactions, and augmented weak-binding samples. Class weights were computed using inverse class frequency and normalized to unit mean.

### 2.5. Feature ablation analysis

To evaluate the relative importance of different structural feature types in guiding model performance, we conducted a systematic ablation analysis. Input features were grouped into three functionally distinct groups: (i) residue-level physicochemical properties, including polarity, charge, isoelectric point, and side-chain size; (ii) edge-level geometric and energetic interactions features, such as inter-residue distance, electrostatic potential, and van der Waals forces; and (iii) surface-derived features, including solvent-accessible surface area, buried surface area, and residue depth.

For each ablation, the model was retrained with one feature group removed, while keeping all other components and hyperparameters fixed. Evaluations were performed on the human TCR-pMHC-I structural dataset, which contains experimentally resolved complexes with annotated binding affinities. The same cross-validation protocol used in the training and testing processes was applied. Performance was assessed using Area Under the ROC Curve (AUC), accuracy, precision, recall, and F1 score to quantify the impact of each feature group on model degradation.

### 2.6. Evaluation of TCRLens performance

To evaluate predictive performance, we employed five rounds of repeated 10-fold cross-validation on the curated human TCR-pMHC structural dataset, ensuring robustness against variability in data partitioning. In each round, data were split in a stratified and group-aware manner to maintain balanced proportions of strong-binding and weak-binding complexes across folds and to prevent structural data leakage between subsets. Specifically, each fold was partitioned into approximately 70% training, 20% validation, and 10% testing sets, with class proportions maintained across all subsets. Model performance was assessed by using standard classification metrics, including Area Under the Receiver Operating Characteristic Curve (AUC), accuracy, precision, recall, and F1 score.

We conducted a comprehensive benchmarking analysis across three prediction tasks. For peptide-MHC binding prediction, TCRLens was evaluated in a restricted setting using only peptide-MHC structural input and compared with existing affinity prediction tools such as NetMHCpan 4.2, CapsNet-MHC, and RPEMHC. For peptide-TCR interaction prediction, we compared TCRLens with peptide-TCR predictors including NetTCR-2.0, PanPep, and TITAN. Lastly, to evaluate full-complex TCR-pMHC prediction, we benchmarked TCRLens against unified models that jointly model all components of the interaction, including ERGO-II, pMTnet, and the structure-based method STAG.

### 2.7. TCRLens generalization to swine and chicken MHC class I systems

To evaluate the cross-species generalizability of TCRLens, we conducted benchmarking experiments using immunogenetic data from *Sus scrofa* (swine) and *Gallus gallus* (chicken), focusing on peptide-MHC Class I interactions relevant to African Swine Fever Virus (ASFV) and various avian pathogens, respectively. Peptide-MHC binding data for swine and chicken curated from experimentally validated binding affinity and eluted ligand assays available in the Immune Epitope Database (IEDB) (Vita *et al.* 2019), comprising 1079 entries for swine (SLA alleles) and 203 entries for chicken (B-F alleles). Peptides with IC50 values below 500 nM were labeled as binders, corresponding to log50k-transformed binding affinity scores greater than 0.426, consistent with the standard threshold used in NetMHCpan. The TCRLens model was initially trained using human peptide-MHC binding data from IEDB and subsequently fine-tuned with species-specific data from swine or chicken to adapt to non-human MHC alleles and peptide repertoires. This two-stage training approach enabled assessment of the model’s ability to generalize to phylogenetically distant species and to peptide-MHC contexts not represented in the original human training data.

Due to the absence of experimentally resolved TCR-pMHC-I structures in swine and chicken, we employed tFold-TCR pipeline to generate complete structural models. This tool constructs 3D TCR-pMHC-I complexes using paired TCR and peptide sequences, along with species-specific MHC allele information. The predicted structures were then processed through the same graph construction and feature extraction pipeline used for human complexes, ensuring consistent input format and feature representation for inference with TCRLens.

To assess full-complex prediction, we evaluated whether TCRLens, trained solely on human TCR-pMHC-I structural data, could generalize to non-human species without additional fine-tuning. In the absence of swine-specific peptide-TCR interaction data, we applied the pre-trained model directly to swine TCR-pMHC-I complexes generated by tFold-TCR. For comparative analysis, we used TITAN, a sequence-based peptide-TCR predictor trained on human BindingDB (Liu *et al.* 2007) data. Because no validated peptide-TCR interaction data exist for swine, we used peptide-SLA binding affinity as a proxy for TCR engagement. Peptides with strong SLA binding (IC50 < 500 nM) were treated as binder. Model performance was evaluated using the area under the ROC curve (AUC), measuring the ability of each model to assign higher interaction likelihood scores to peptides with strong SLA binding affinity.

## 3. Results

### 3.1. Structural validation of predicted TCR-pMHC-I complexes

Analysis of the structural dataset revealed consistent and biologically plausible characteristics of TCR-pMHC-I interfaces at both the graph and interaction-point levels (Supplementary Tables S2 and S3). At the graph level, residue-based interface graphs contained a moderate number of nodes and edges, reflecting compact yet well-connected interaction networks spanning peptide, MHC, and TCR residues. Graphs corresponding to binding complexes exhibited larger size, more densely connected than those derived from weak-binding interactions, exhibiting increased edge density and higher average node degrees. In addition, graphs associated with binding interactions showed slightly shorter mean inter-residue distances consistent with more extensive and tightly packed interfaces.

At the interaction-feature level, strong-binding complexes exhibited subtle but reproducible physicochemical differences relative to weak-binding complexes. Strong-binding interactions were characterized by marginally shorter mean inter-residue distances, indicative of closer residue packing. Electrostatic interaction potentials were lower in strong-binding interactions, while van der Waals interactions were more favorable, suggesting stronger and more stable interfacial contacts. Furthermore, strong-binding complexes showed larger interface surface areas and substantially higher numbers of salt bridges, hydrogen-bonding residues, and interface-specific contacts, accompanied by more favorable stability and solvation energy profiles.

To further dissect interaction patterns at finer resolution, atom-level contacts were aggregated into residue-level statistics for peptide-TCR interface. Both TCRα and TCRβ chains contributed substantially to peptide recognition, albeit with distinct quantitative patterns. Peptide-TCRβ interfaces involved a greater number of atomic contacts and a larger set of participating TCR residues than peptide-TCRα interfaces, consistent with the dominant role of the β chain in peptide engagement reported in structural immunology studies (Rudolph *et al.* 2006). In contrast, the number of peptide residues involved in contacts was comparable between α and β chains, indicating similar peptide coverage. Mean interatomic contact distances were similar across peptide-TCRα and peptide-TCRβ interfaces, reflecting comparable geometric packing despite differences in interface size.

**Figure 2:**
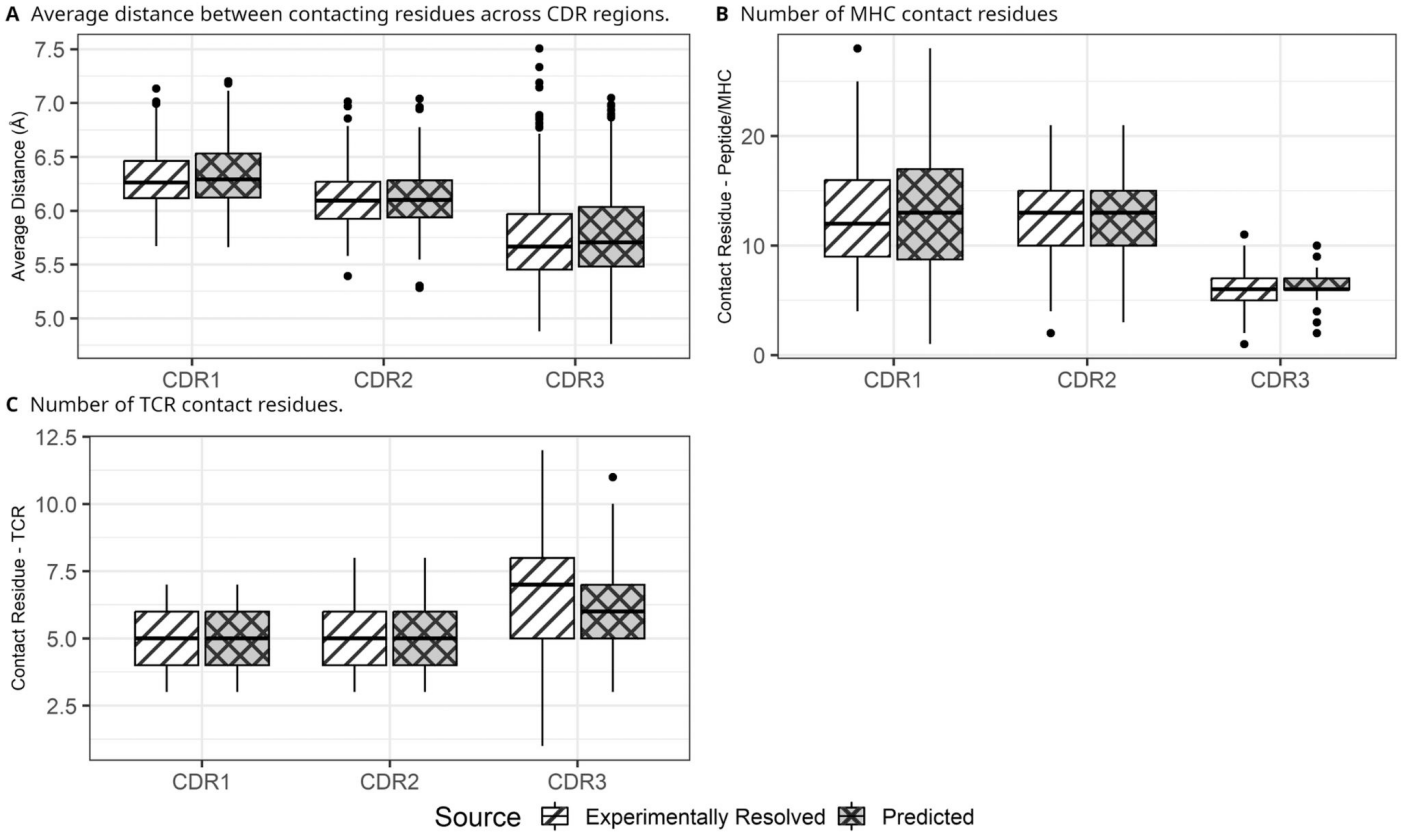
Comparison of interaction patterns between experimentally resolved and predicted TCR-pMHC-I structures. (A) Mean distance between contacting residues across CDR regions. (B) Number of contact residues on the MHC (for CDR1 and CDR2) or peptide (for CDR3). (C) Number of contact residues on the TCR. Contact residues were defined using the DeepRank2 pipeline, with contacts identified when any heavy atom of a residue was within 8 Å of any heavy atom of the interacting partner.

Having established the structural characteristics of experimental resolved complex, we next evaluated whether predicted TCR-pMHC-I complexes preserve comparable interaction patterns. Residue-level contact graphs derived from tFold-TCR-predicted models were directly compared with those constructed from experimentally resolved structures, sharing the identical TCR and pMHC-I sequences (see Section 2: Validation of Predicted TCR-pMHC-I Structures). As shown in Fig. 2, we analyzed three edge-based metrics across CDR regions: (i) average distance between contacting residues, (ii) number of peptide or MHC contact residues, and (iii) number of TCR contact residues. Predicted structures from tFold-TCR (Wu *et al.* 2025) showed slightly shorter average distances and modestly higher contact counts across CDR1, CDR2, and CDR3 of both chains, in peptide-TCR and MHC-TCR interfaces. However, these differences were not statistically significant (two-sided Mann-Whitney *U* test, *P* > .05). In addition, interface properties of predicted complexes were systematically compared against experimentally resolved structures using a non-parametric statistical framework. Differences in performance metric values across models were evaluated using the Mann-Whitney *U* test, with effect sizes estimated to assess the practical magnitude of observed differences. As summarized in Supplementary Table S4, predicted complexes exhibited distributions largely overlapping with those of experimental structures, and effect sizes were predominantly negligible or small. Importantly, no systematic bias toward interfaces characteristic of crystal packing artefacts was observed. Together, these results indicate that, despite minor geometric variations, predicted complexes preserve the coarse-grained residue contact patterns characteristic of experimentally resolved TCR-pMHC-I interfaces.

### 3.2. Analysis of structural feature contributions to TCRLens performance

**Figure 3:**
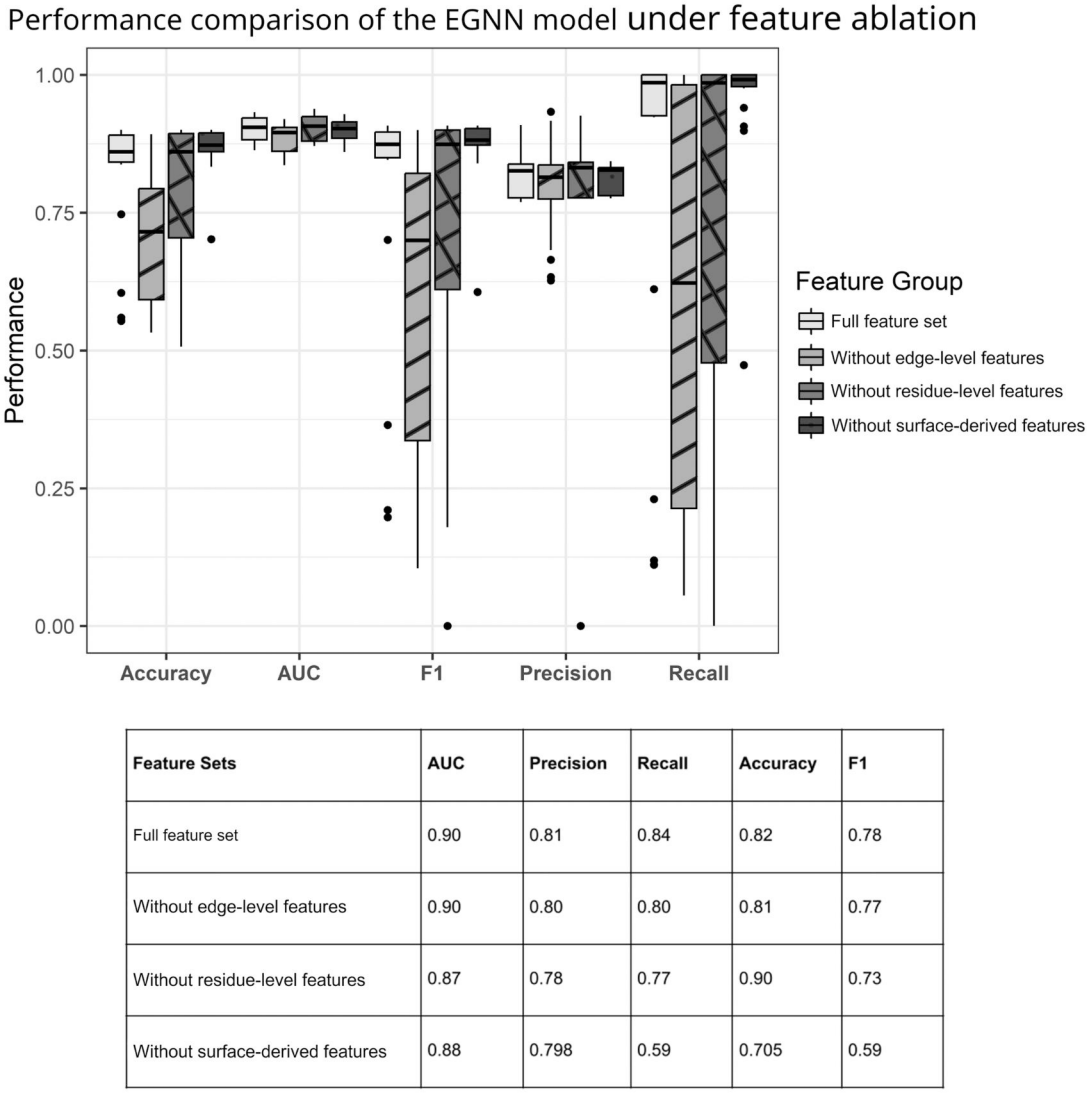
Performance comparison of the EGNN model under ablation of structural feature groups. Model performance was evaluated using repeated cross-validation framework. Box plots summarize the distributions of Accuracy, AUC, F1 score, Precision, and Recall across five rounds of repeated 10-fold cross-validation. In each box plot, the central line denotes the median, the box represents the interquartile range (25th to 75th percentiles), and the whiskers indicate the full range of values observed across cross-validation runs.

We conducted an ablation study to quantify the contribution of different structural feature groups to model performance, using the experimental dataset and evaluation metrics described in the Feature Ablation Analysis in Section 2. Input features were grouped into three categories: residue-level physicochemical properties, edge-level features, and surface-derived features. The results (Fig. 3) demonstrate differential effects of each feature group on predictive performance:

- Residue-level features: Removal of residue-level physicochemical features resulted in a consistent reduction in performance, with AUC decreasing from 0.90 to 0.86, precision from 0.81 to 0.78, accuracy from 0.82 to 0.79, and F1 score from 0.78 to 0.73, while recall remained largely unchanged (0.838–0.840).
- Edge features: Exclusion of edge-level features produced the most substantial performance decline, with AUC decreasing to 0.88, precision to 0.79, recall to 0.59, accuracy to 0.70, and F1 score to 0.58.
- Surface-derived features: Removal of surface-derived features had a comparatively modest effect, with AUC decreasing to 0.90, precision to 0.80, recall to 0.80, accuracy to 0.81, and F1 score to 0.77.

Taken together, the ablation results indicate that edge-level interaction features contribute most strongly to predictive performance, as their removal results in the largest declines across evaluation metrics, particularly recall and F1 score. Residue-level physicochemical features also play a substantial role, with consistent reductions in AUC, precision, and accuracy observed upon exclusion. In contrast, surface-derived features exhibit more limited, metric-specific effects, primarily influencing recall-related measures.

### 3.3. Validating VAE-GAN-generated weak-binding TCR-pMHC-I interfaces

To mitigate the scarcity of experimentally verified weak-affinity binding TCR-pMHC-I complexes, the training dataset was augmented with structure-guided weak-binding decoys, generated using the Graph VAE-GAN (see Section 2: Generative Augmentation of Weak-Binding TCR-pMHC-I Interfaces). The quality of generated decoys were evaluated along two dimensions: (i) local feature fidelity, quantified by KL divergence between experimentally derived and augmented node- and edge-level feature distributions, and (ii) global structural coherence, assessed using MMD with the Weisfeiler-Lehman subtree kernel.

As summarized in Supplementary Table 5, node-level features, including polarity, residue mass, and solvent accessibility, exhibited low KL divergence (predominantly < 0.001), indicating close agreement between experimentally validated and generated weak-binding interfaces at the feature level. In contrast, edge-level features showed higher divergence, particularly for electrostatic potential (5.61) and inter-residue distance (3.68), reflecting increased variability in fine-grained spatial and physicochemical interactions. The low MMD score (3.96 × 10^−6^) indicates that generated decoys retained globally coherent connectivity patterns comparable to those of experimentally resolved TCR-pMHC-I complexes. Analysis of the latent embedding space further revealed that GAN-augmented weak-binding samples formed a distinct cluster separated from experimentally validated strong-binding complexes (Supplementary Fig. S3), indicating that the generated decoys occupied regions of the learned interface manifold, distinct from strong-binding interaction states. These results demonstrate that the generated decoys expand the diversity of weak-binding samples while maintaining structural coherence, supporting improved discrimination in downstream classification tasks, particularly for interfaces with structural similarity.

### 3.4. Comparative performance of TCRLens and prior models

To benchmark TCRLens against existing tools, we evaluated its performance across three predictive tasks: (i) peptide-MHC binding prediction, assessing the identification of peptides presented by MHC molecules; (ii) peptide-TCR recognition, evaluating the TCR specificity for antigenic peptides; and (iii) full-complex TCR-pMHC-I interaction prediction, which involves modeling the complete molecular interface among TCR, peptide, and MHC components. Model performance was first assessed using five rounds of repeated 10-fold cross-validation on a curated dataset of human TCR-pMHC-I structural complexes containing both strong-binding and weak-binding samples (see Section 2). TCRLens achieved a mean AUC of 0.90 ± 0.04 across validation folds as shown in the Supplementary Fig. S4. In contrast, the same model trained without generative augmentation yielded a substantially lower mean AUC of 0.78, highlighting the contribution of weak-binding interface augmentation to predictive performance. To further assess whether the resulting prediction scores capture latent affinity-related information, we conducted a post-hoc analysis relating model scores to experimentally measured affinities using a Gaussian mixture model with two linear components and 500 rounds of balanced subsampling, revealing a reproducible association between predicted scores and experimentally measured binding affinities (mean *R*^2^ ≈ 0.7), indicating that TCRLens implicitly encodes affinity-related trends beyond binary classification.

**Figure 4:**
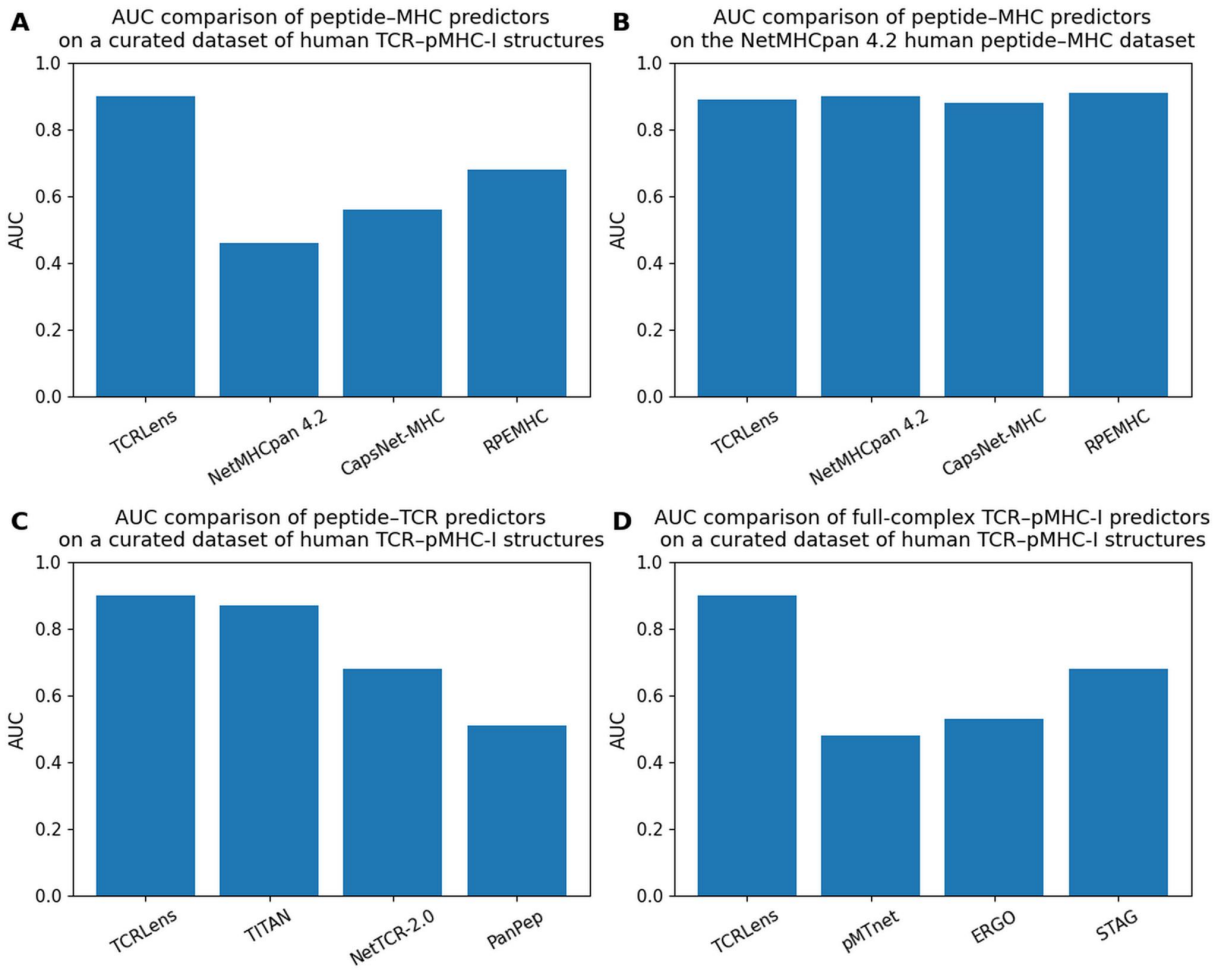
Performance comparison of TCRLens with existing predictors across multiple TCR-pMHC-I-related tasks. (A) Area under the ROC curve (AUC) for peptide-MHC binding prediction evaluated on a curated dataset of human TCR-pMHC-I structures. (B) Peptide-MHC binding performance evaluated on the NetMHCpan 4.2 human peptide-MHC dataset. (C) Peptide-TCR recognition performance evaluated on a curated dataset of human TCR-pMHC-I structures. (D) Full-complex TCR-pMHC-I interaction prediction performance evaluated on a curated dataset of human TCR-pMHC-I structures.

In the peptide-MHC benchmark (Fig. 4A), evaluated on a curated dataset of human TCR-pMHC-I structural complexes, TCRLens achieved an AUC of 0.90, exceeding the performance of NetMHCpan 4.2 (AUC = 0.46), CapsNet-MHC (0.56), and RPEMHC (0.68). To further assess peptide-MHC binding performance in a standardized setting, TCRLens was evaluated on the NetMHCpan 4.2 dataset, achieving an AUC of 0.906. Under the same evaluation protocol, NetMHCpan 4.2, CapsNet-MHC, and RPEMHC achieved AUC values of 0.901. and 0.887 and 0.892 respectively, indicating comparable performance despite TCRLens being trained for full-complex modeling (Fig. 4B). TCRLens also demonstrated superior performance, in the peptide-TCR recognition task, achieving an AUC of 0.90 and surpassing TITAN (AUC = 0.87), NetTCR-2.0 (AUC = 0.68), and PanPep (AUC = 0.51) (Fig. 4C). Unlike these models, which primarily rely on sequence-based features or motif matching, TCRLens leverages an EGNN-based architecture that captures the spatial relationships essential for precise binding prediction.

In the TCR-peptide-MHC interaction full-complex setting (Fig. 4D), TCRLens outperformed unified models such as pMTnet (AUC = 0.48), ERGO II (0.53), and STAG (0.68). Although STAG incorporates structural information via graph neural networks, it represents residue interactions mainly using static physicochemical descriptors of amino acids and distance-based spatial proximity. In contrast, TCRLens learns geometry-aware representations through equivariant message passing and integrates explicit physicochemical and structural attributes of the interface across five biologically relevant interaction zones: peptide-MHC, peptide-TCRα, peptide-TCRβ, MHC-TCRα, and MHC-TCRβ. This comprehensive representation enables the model to capture inter-chain cooperativity and the full topology of the TCR-pMHC-I interface.

### 3.5. Binding across species: TCRLens performance in swine and chicken

To evaluate the cross-species generalizability of TCRLens, we assessed its performance on peptide-MHC Class I binding prediction in two non-human species: Sus scrofa (swine, SLA) and Gallus gallus (chicken, B-F). These species are relevant for veterinary immunology and zoonotic disease research. For each species, we fine-tuned a model pre-trained on human peptide-MHC-I complexes using experimentally validated binding data curated from the IEDB (Vita *et al.* 2019).

**Figure 5:**
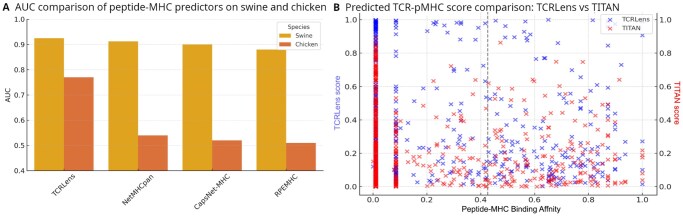
Performance evaluation on non-human peptide-MHC complexes. (A) Comparison of AUC values for peptide-MHC predictors evaluated on swine (SLA) and chicken MHC peptide-binding datasets. (B) Comparison of predicted TCR-pMHC-I binding scores produced by TCRLens and TITAN. The *x*-axis denotes ground-truth peptide-MHC binding affinity derived from NetMHCpan annotations, while the *y*-axis reports the corresponding predicted binding scores pfrom each model, illustrating their relationship with experimentally derived binding strength. The dashed line indicates the NetMHCpan-defined threshold separating strong- and weak-binding interactions and serves as a reference boundary. TITAN was selected for comparison in this panel as it achieved the highest AUC among the evaluated baseline methods in the benchmarking experiments.

As an initial benchmark, we trained TCRLens to model only the peptide-MHC interface, excluding TCR input to enable direct comparison with existing MHC-focused models. Swine-specific peptide-MHC-I binding data were derived from the NetMHCpan 4.2 training dataset (see Section 2: TCRLens Generalization to Swine and Chicken MHC Class I Systems). As shown in Fig. 5A, TCRLens achieved the highest AUC (0.92), outperforming NetMHCpan 4.2 (0.91), CapsNet-MHC (0.90), and RPEMHC (0.88). These results indicate that TCRLens delivers robust predictive performance across phylogenetically diverse MHC-I alleles, even when provided with partial structural inputs.

We then extended the evaluation to *Gallus gallus* (chicken), focusing on peptide binding to Class I MHC alleles. TCRLens again demonstrated superior performance, achieving an AUC of 0.77 substantially outperforming NetMHCpan 4.2 (0.54), CapsNet-MHC (0.52), and RPEMHC (0.51). These results confirm that TCRLens maintains strong predictive power across phylogenetically diverse MHC alleles, even when limited to partial structural inputs and non-human species. Importantly, TCRLens correctly identified several peptide-SLA binding interactions that were misclassified by NetMHCpan 4.2. For instance, peptides such as RSVWIPGRW, MTRGLLGSY, and VSDGGPNLY were assigned weak-binding scores (< 0.5) by NetMHCpan 4.2 but predicted to form strong-binding interactions (> 0.5) by TCRLens (Table 1). These peptides also classified as strong binding interactions by TITAN, a TCR-aware model, further supporting their immunogenic potential. These findings highlight TCRLens’s capacity to recover likely true positives that are missed by conventional MHC-centric predictors, understanding its utility for epitope discovery across species.

**Table 1:** Comparison of peptide-SLA binding predictions across models.

Peptide	Binding affinity	TCRLens prediction score	NetMHCpan prediction score
RSVWIPGRW	0.45	0.57	0.11
VSDGGPNLY	0.91	0.53	0.39
MTRGLLGSY	0.43	0.51	0.33
ESLLHQASW	0.51	0.85	0.15
MTRRRVLSV	0.56	0.74	0.49

SLA Binding Affinity values are derived from NetMHCpan training data. Peptides with NetMHCpan affinity scores > 0.426 are considered binders.

To further prove cross-species transferability at the TCR-pMHC level, we applied TCRLens, trained exclusively on human TCR-pMHC-I structural data, to a swine-specific setting. Peptides with known SLA binding affinities were used to construct full pMHC complexes, and paired with TCRs for prediction, despite the absence of experimentally validated peptide-TCR interactions in swine. Peptides with normalized SLA binding scores above 0.426 (corresponding to IC50 < 500 nM on the log50k scale) were labeled as putative binding interactions. Under this indirect evaluation framework, TCRLens achieved a higher AUC (0.56) than TITAN (0.49, Fig. 5B), indicating improved sensitivity of TCRLens when extrapolating to previously unseen MHC alleles. Although absolute AUC values were modest, as expected given that peptide-SLA binding affinity is only a proxy for TCR recognition–the relative performance gain suggests that structural interaction patterns learned from human TCR-pMHC-I complexes are partially conserved across species.

These results underscore the robustness of TCRLens for cross-species immune modeling. By leveraging full 3D interface representations, it not only delivers accurate peptide-MHC predictions but also demonstrates promising generalizability to uncharacterized MHC alleles in swine and chicken. These capabilities make TCRLens a valuable tool for veterinary immunology, zoonotic disease research and translational vaccine development. To support reproducibility and broad accessibility, TCRLens prediction scores for cytotoxic T lymphocyte (CTL) responses are publicly available through the EpiBase platform (https://epibase.tbrcnetwork.org/), covering both NetMHCpan-predicted peptide candidates and experimentally validated epitopes curated by the Immune Epitope Database (IEDB) (Vita *et al.* 2019).

## 4. Discussion

In this study, we introduced TCRLens, a structure-aware deep learning framework to predict TCR-pMHC-I interactions by integrating multi-scale structural representations, equivariant graph neural networks (EGNNs), and structure-guided decoys for data augmentation. Across multiple prediction tasks—peptide-MHC binding, peptide-TCR recognition, and full-complex TCR-pMHC-I binding—TCRLens consistently outperformed both task-specific and unified baseline models. These results highlight the importance of explicitly modeling three-dimensional interface geometry in immunoinformatics.

In contrast to existing approaches such as NetTCR-2.0, PanPep, and ERGO-II, which rely on sequence embeddings and typically focus on CDR3-peptide contacts, TCRLens captures the full TCR-pMHC interface. It encodes five biologically relevant contact zones, including the often-overlooked MHC-CDR1/CDR2 interactions. Compared to STAG, a structure-aware graph neural network for TCR-pMHC interaction analysis, TCRLens incorporates a broader set of residue-level features, including physicochemical properties, geometric descriptors, and surface-accessibility. Through its equivariant message-passing mechanism, the model updates both node features and spatial coordinates, enabling the learning of rotation-invariant biophysically grounded interaction patterns.

Importantly, TCRLens demonstrated strong cross-species generalization in peptide-MHC binding prediction. When fine-tuned on peptide-MHC Class I data from Sus scrofa (SLA), the model achieved performance comparable to or exceeding state-of-the-art tools like NetMHCpan. Furthermore, when applied to MHC Class I alleles from Gallus gallus (chicken), TCRLens continued to outperform baseline models, albeit with reduced AUC. This performance drop likely reflects the limited availability and diversity of experimentally validated chicken MHC binding data. Nonetheless, these results demonstrate the model’s adaptability to non-human immunogenetic context and supporting its potential in species-specific epitope prediction.

In the more challenging task of TCR-pMHC-I binding prediction, TCRLens was trained exclusively on human structural data due to the scarcity of experimentally resolved TCR-pMHC complexes in non-human species. Despite this limitation, TCRLens demonstrated effective generalization to swine-derived pMHC complexes, and outperformed TITAN, a leading sequence-based model trained on human BindingDB data, by assigning higher scores to putative TCR binders. These findings suggest that TCRLens captures transferable structural features that generalize across species, highlighting its potential for cross-species immune modeling, particularly in veterinary and zoonotic research contexts.

Beyond binding prediction, TCRLens offers a flexible platform for integration into immune modeling workflows. Its modular design supports multiple input configurations, enabling structure-aware predictions for peptide-MHC, peptide-TCR, or full-complex TCR-pMHC-I interactions. For example, TCRLens can be deployed as a structure-based re-ranking component within T cell epitope discovery pipelines, prioritizing candidate peptides not only by MHC binding affinity but also by their inferred propensity to engage TCRs. This dual-layer screening strategy has the potential to improve the identification of truly immunogenic epitopes, which are more likely to elicit effective T cell responses. Such capabilities are particularly relevant for vaccine development targeting infectious diseases and emerging pathogens. Moreover, the observed generalization of TCRLens across MHC alleles and species suggests broader applicability to diverse mammalian systems with comparable immunogenetic architectures.

Despite these strengths, several limitations remain, primarily reflecting constraints in current data availability. Experimentally validated TCR-pMHC-I binding data are limited in scope, with particularly sparse coverage of true non-binders and non-human species. Moreover, existing structural resources, including ATLAS and TCR3d are strongly biased toward human-derived positively interacting complexes with relatively high-binding affinities, thereby restricting data diversity and the range of interaction modes represented. As a result, this study necessarily relies on weak-affinity binding complexes and structure-guided decoys as proxies for negative examples. Although the VAE-GAN-generated decoys are designed to be structurally plausible, they may not fully recapitulate the biological heterogeneity of genuine non-binding or transient interfaces, and subtle modeling biases cannot be ruled out. In addition, the limited availability of experimentally resolved non-human TCR-pMHC-I structures constrains direct validation of cross-species TCR recognition. Finally, TCRLens currently focuses on MHC class I-restricted complexes, limiting its applicability to CD8^+^ T cell responses. Extending the framework to MHC class II-mediated CD4^+^ T cell recognition will require explicit modeling of longer peptides, class-specific binding geometries, and the incorporation of additional high-quality structural data as such resources become available.

All reported evaluations were conducted using repeated cross-validation framework on a curated structural dataset. While this approach provides a statistically robust assessment of internal generalization within the available data distribution, it may yield optimistic performance estimates compared to real-world deployment, where data distributions differ substantially and experimentally resolved structures are often unavailable. To partially address this limitation, we further evaluated TCRLens on predicted TCR-pMHC structures, reflecting practical application scenarios where experimentally resolved complexes are unavailable.

Independent benchmarking of full-complex TCR-pMHC-I interaction models remains inherently challenging. Large-scale external datasets, such as mass spectrometry-derived MHC ligandome resources, are primarily limited to peptide-MHC binding and lack paired TCR information, precluding direct validation of full-complex TCR-pMHC-I predictions. Consequently, independent evaluation in this study was restricted to peptide-MHC binding tasks using NetMHCpan dataset, whereas TCR-pMHC-I binding performance was predominatly assessed through cross-validation on curated structural datasets. This limitation is shared by many current TCR modeling studies and highlights the need for standardized benchmarking resources that include both strong- and weak-binding TCR-pMHC interactions.

As a structure-aware framework, TCRLens requires three-dimensional models of TCR, peptide, and MHC molecules. In many applications, such structures are not experimentally available and must be obtained through homology modeling or structure prediction. While recent advances in protein structure prediction have substantially lowered this barrier, modeling inaccuracies may propagate into interface representations and affect prediction reliability. As such, TCRLens is best suited for applications where structural models of sufficient quality can be generated, such as structure-guided epitope prioritization, mechanistic studies, or post hoc re-ranking rather than large-scale sequence-only screening.

In summary, this work demonstrates the value of structure-informed, geometry-aware modeling for TCR-pMHC-I interaction prediction. By bridging structural immunology and deep learning, TCRLens provides a robust foundation for next-generation tools in epitope discovery, personalized immunotherapy, and cross-species immune modeling. Continued progress will depend on the expansion of high-quality structural datasets, particularly those including verified weak-binding and non-human complexes, to further improve model generalization and biological fidelity.

## Supplementary Material

vbag066_Supplementary_Data

## Data Availability

The code underlying this article are available in GitHub, at https://github.com/paopitsiri/TCRLens.
