# Supplementary material for: TCRLens: structure-aware equivariant graph learning for TCR-pMHC-I recognition and immunogenic epitope discovery
Source: Bioinform Adv. 2026 Feb 24;6(1):vbag066. doi: 10.1093/bioadv/vbag066 (PMC13012892; doi:10.1093/bioadv/vbag066)
Supplement: vbag066_Supplementary_Data [file vbag066_supplementary_data.zip › supplementary material-final.docx]

**Supplementary data**

TCRLens: Structure-Aware Equivariant Graph Learning for TCR-pMHC-I Recognition and Immunogenic Epitope Discovery

Paopit Siriarchawatana^1,2^, Supawadee Ingsriswang^1,2,^*, Challika Kaewborisuth^2,3^, Anan Jongkaewwattana^2,3^

^1^Microbial Systems and Computational Biology Research Team, Thailand Bioresource Research Center (TBRC)

^2^Virology and Vaccine Technology Research Team, Veterinary Health Innovation and Management Research Group

^3^National Center for Genetic Engineering and Biotechnology (BIOTEC), Thailand

*Corresponding author. National Center for Genetic Engineering and Biotechnology, 113 Thailand Science Park, Phahonyothin Road, Khlong Nueng, Khlong Luang, Pathum Thani 12120 Thailand, E-mail address: supawadee@biotec.or.th, Tel: +66-2564-6700, Fax: +66-2564-6701-5

Associate Editor: Kieran Campbell

**Supplementary Table S1:** Node and edge features for residue-level graph representation of TCR-pMHC-I interfaces

| **Feature Type** | **Feature Group** | **Feature Name** | **Description** |
| --- | --- | --- | --- |
| Node | residue properties | Amino acid identity | Encoded amino acid identity of the residue |
| Node | residue properties | Side-chain polarity | Side chain polarity indicating hydrophobicity or hydrophilicity |
| Node | residue properties | Side-chain volume (normalized) | Normalized side chain volume (relative to glycine) |
| Node | residue properties | Residue molecular mass | Molecular mass of the amino acid residue |
| Node | residue properties | Residue size | The number of non-hydrogen atoms in the side chain |
| Node | residue properties | Net residue charge | Net residue charge at physiological pH |
| Node | residue properties | Residue isoelectric point (pI) | Isoelectric point of the residue |
| Node | surface accessibility features | Residue depth from molecular surface | Residue depth (Å) from the molecular surface, computed via the residue depth method (Chakravarty & Varadarajan, 1999; Pintar et al., 2003). |
| Node | surface accessibility features | Half-Sphere Exposure (HSE) | Half-Sphere Exposure (local packing density) via the HSE method (Hamelryck, Bioinformatics, 2005). |
| Node | surface accessibility features | Solvent Accessible Surface Area (SASA) | Solvent Accessible Surface Area (Å²) via Shrake-Rupley algorithm |
| Node | surface accessibility features | Buried Surface Area (BSA) | Buried Surface Area (Å²) is the loss of solvent-accessible surface that occurs upon complex formation, computed using the same Shrake-Rupley for the isolated partners and the complex. |
| Edge | edge features | distance | Euclidean distance between residue centroids |
| Edge | edge features | Same-chain indicator | Binary indicator whether both residues are from the same chain |
| Edge | edge features | Covalent bond indicator | Boolean flag indicating covalent bond between the residues |
| Edge | edge features | Electrostatic interaction potential | Electrostatic potential between two nodes, calculated using interatomic distances and charges of each atom |
| Edge | edge features | Van der Waals interaction potential | Van der Waals potential between two nodes, calculated using interatomic distance |

**Supplementary Table S2:** Summary statistics of graph structural properties for positive and negative TCR-pMHC-I complexes.

| Graph Properties | Strong-Binding Dataset (mean ± SD) | Weak-Binding Dataset (mean ± SD) |
| --- | --- | --- |
| Number of nodes | 134.586 ± 18.084 | 131.868 ± 20.83 |
| Number of edges | 1501.915 ± 278.295 | 1470.868 ± 321.466 |
| Average degree | 22.121 ± 1.542 | 22.038 ± 1.794 |
| Residue molecular mass | 118.688 ± 2.857 | 118.118 ± 3.219 |
| Residue Size | 4.431 ± 0.205 | 4.389 ± 0.23 |
| Residue depth from molecular surface | 3.489 ± 0.21 | 3.429 ± 0.228 |
| Residue isoelectric point (pI) | 6.117 ± 0.076 | 6.112 ± 0.083 |
| Buried Surface Area (BSA) | 12.199 ± 10.022 | 13.223 ± 11.693 |
| Solvent Accessible Surface Area (SASA) | 25.322 ± 1.57 | 25.906 ± 1.552 |
| Electrostatic interaction potential | 2.843 ± 5.151 | 4.007 ± 5.724 |
| Van der Waals interaction potential | -0.243 ± 0.247 | -0.174 ± 0.276 |

**Supplementary Table S3:** Interface interaction and energetic properties of positive and negative TCR-pMHC-I complexes

| Properties | Strong-Binding Dataset (mean ± SD) | Weak-Binding Dataset (mean ± SD) |
| --- | --- | --- |
| Interface surface area | 662.3 ± 318.0 | 369.8 ± 266.6 |
| Number of salt bridges | 2.09 ± 2.73 | 0.31 ± 0.77 |
| Number of salt-bridge residues | 2.06 ± 2.36 | 0.41 ± 0.89 |
| Number of specific interface residues | 9.30 ± 6.01 | 5.02 ± 3.90 |
| Stability energy | −10.00 ± 7.60 | −5.19 ± 4.20 |
| Number of hydrogen-bond residues | 7.98 ± 5.70 | 4.75 ± 3.85 |
| Solvation energy | −6.80 ± 5.76 | −3.62 ± 3.20 |
| Number of hydrogen bonds | 5.46 ± 4.60 | 3.28 ± 3.04 |

**Supplementary Table S4:** Comparison of interface-level properties between experimentally resolved and predicted TCR-pMHC-I complexes

| **Interface** | **Properties** | **Experimental Resolved Structure** | **Predicted Structure** | **Experimental Resolved Structure** | **Predicted Structure** | **p-value** | **cliffs delta** | **effect size** |
| --- | --- | --- | --- | --- | --- | --- | --- | --- |
| MHC-Peptide | Interface surface area | 849.78 | 833.41 | 849.12 | 831.59 | 0.01 | 0.17 | small |
| MHC-Peptide | Number of hydrogen bonds | 12.00 | 11.00 | 12.03 | 11.38 | 0.04 | 0.13 | negligible |
| MHC-Peptide | Number of salt bridges | 2.00 | 1.50 | 2.06 | 2.02 | 0.40 | -0.05 | negligible |
| MHC-Peptide | Solvation energy | -10.14 | -10.26 | -9.86 | -10.05 | 0.60 | 0.03 | negligible |
| MHC-Peptide | Stability energy | -16.01 | -16.14 | -15.97 | -15.86 | 0.98 | 0.00 | negligible |
| MHC-TCRα | Interface surface area | 385.42 | 392.00 | 377.81 | 389.92 | 0.62 | -0.03 | negligible |
| MHC-TCRα | Number of hydrogen bonds | 3.00 | 2.00 | 3.29 | 2.72 | 0.03 | 0.14 | negligible |
| MHC-TCRα | Number of salt bridges | 1.00 | 0.00 | 1.27 | 0.89 | 0.01 | 0.15 | small |
| MHC-TCRα | Solvation energy | -1.73 | -1.19 | -1.57 | -1.31 | 0.11 | -0.11 | negligible |
| MHC-TCRβ | Stability energy | -3.47 | -2.85 | -3.51 | -2.85 | 0.00 | -0.21 | small |
| MHC-TCRβ | Interface surface area | 366.77 | 376.67 | 363.46 | 374.87 | 0.38 | -0.06 | negligible |
| MHC-TCRβ | Number of hydrogen bonds | 3.00 | 2.00 | 2.97 | 2.25 | 0.00 | 0.20 | small |
| MHC-TCRβ | Number of salt bridges | 1.00 | 0.00 | 1.21 | 0.99 | 0.03 | 0.13 | negligible |
| MHC-TCRβ | Solvation energy | -1.57 | -2.02 | -1.84 | -2.04 | 0.32 | 0.07 | negligible |
| MHC-TCRβ | Stability energy | -3.33 | -3.34 | -3.61 | -3.41 | 0.64 | -0.03 | negligible |
| peptide-TCRα | Interface surface area | 156.89 | 128.67 | 156.39 | 128.27 | 0.00 | 0.29 | small |
| peptide-TCRα | Number of hydrogen bonds | 2.00 | 1.00 | 2.08 | 0.97 | 0.00 | 0.37 | medium |
| peptide-TCRα | Number of salt bridges | 0.00 | 0.00 | 0.20 | 0.13 | 0.21 | 0.04 | negligible |
| peptide-TCRα | Solvation energy | -1.05 | -0.98 | -1.33 | -1.23 | 0.77 | -0.02 | negligible |
| peptide-TCRβ | Stability energy | -2.04 | -1.53 | -2.33 | -1.71 | 0.00 | -0.26 | small |
| peptide-TCRβ | Interface surface area | 189.42 | 185.27 | 179.42 | 181.79 | 0.77 | -0.02 | negligible |
| peptide-TCRβ | Number of hydrogen bonds | 3.00 | 1.00 | 2.74 | 1.58 | 0.00 | 0.40 | medium |
| peptide-TCRβ | Number of salt bridges | 0.00 | 0.00 | 0.57 | 0.17 | 0.12 | 0.06 | negligible |
| peptide-TCRβ | Solvation energy | -1.19 | -1.68 | -1.28 | -1.88 | 0.00 | 0.24 | small |
| peptide-TCRβ | Stability energy | -2.88 | -2.71 | -2.71 | -2.64 | 0.59 | -0.04 | negligible |

**Supplementary Table S5:** Kullback-Leibler (KL) divergence of feature distributions between experimentally resolved and augmented negative samples

| **Feature Type** | **Feature Name** | **KL Divergence** |
| --- | --- | --- |
| Node | Amino acid identity | 0.000583 |
|  | Side-chain polarity | 0.001778 |
|  | Side-chain volume (normalized) | 0.000161 |
|  | Residue molecular mass | 0.000079 |
|  | Net residue charge | 0.000831 |
|  | Residue isoelectric point (pI) | 0.000094 |
|  | Residue depth from molecular surface | 0.000711 |
|  | Half-Sphere Exposure (HSE) | 0.000375 |
|  | Solvent Accessible Surface Area (SASA) | 0.000001 |
| Edge | Buried Surface Area (BSA) | 3.681074 |
|  | Inter-residue distance | 2.017754 |
|  | Same-chain indicator | 5.610991 |
|  | Covalent bond indicator | 2.351296 |

**Supplementary Figure**


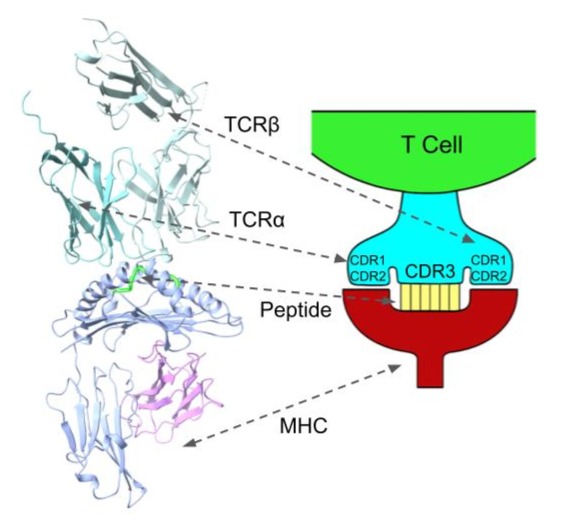


**Figure S1** TCR-pMHC-I interface organization modeled by TCRLens.
TCRLens models interactions across five biologically defined interface zones: peptide-MHC, peptide-TCRα, peptide-TCRβ, MHC-TCRα, and MHC-TCRβ.

**
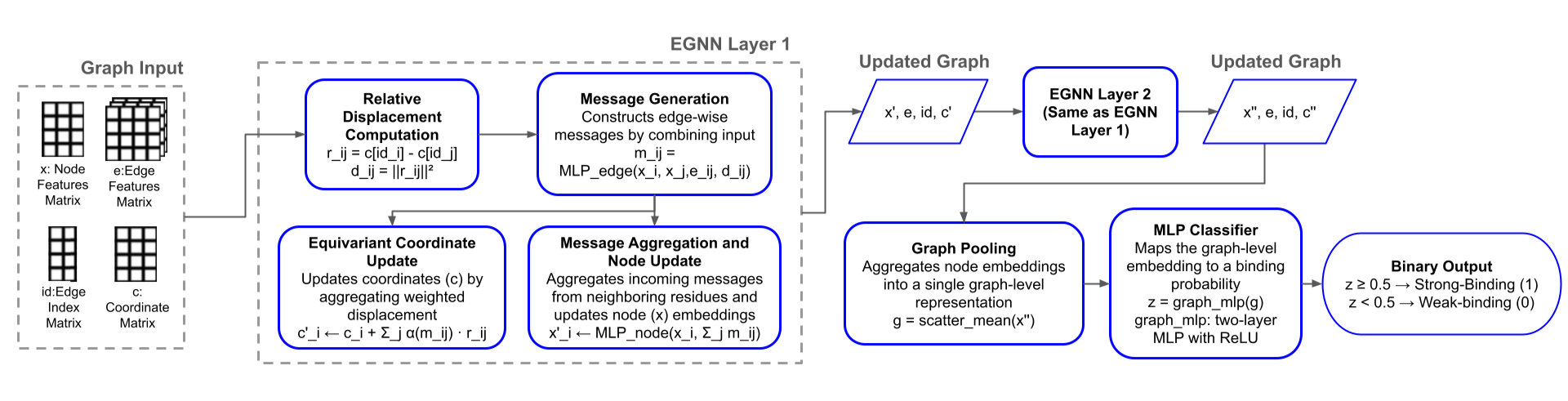
**

**Figure S2** EGNN-based workflow for graph-level TCR-pMHC-I recognition.

**
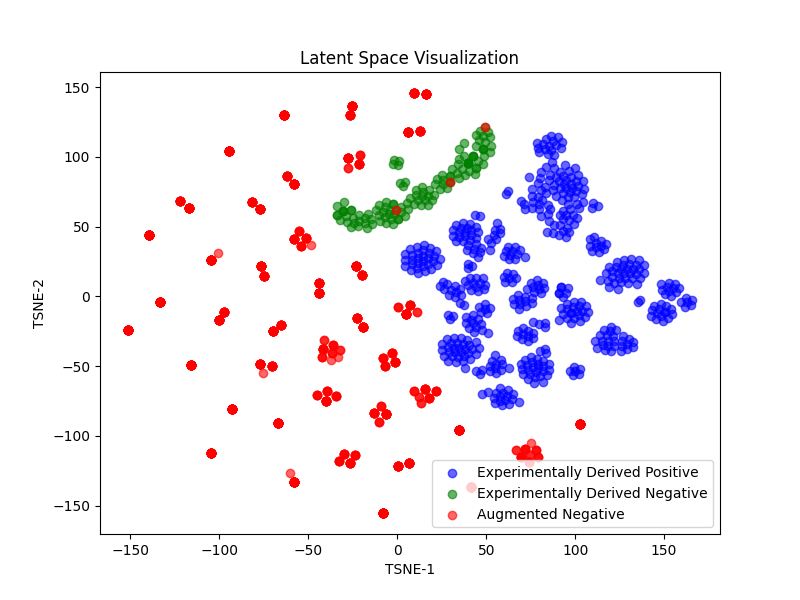
**

**Figure S3** Latent space separation between experimentally resolved and augmented TCR-pMHC-I complexes.

Two-dimensional visualization of the learned latent representations generated using t-distributed stochastic neighbor embedding (t-SNE). The two axes (TSNE-1 and TSNE-2 ) correspond to the dimensions of the nonlinear embedding


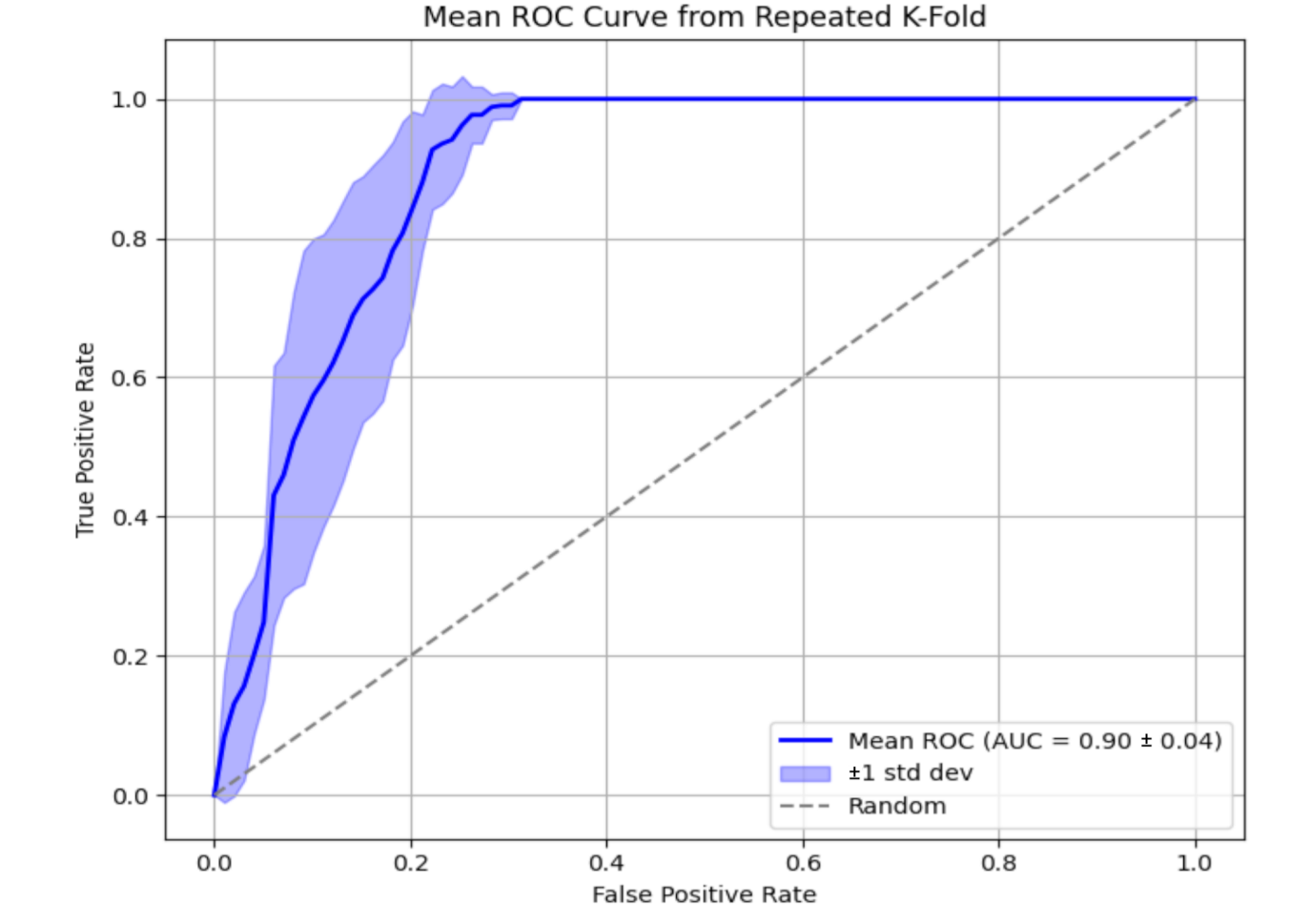


**Figure S4** ROC curves of TCRLens performance on a curated dataset of human TCR-pMHC-I structures.
